# Fight Hard or Die Trying: Current Status of Lipid Signaling during Plant–Pathogen Interaction

**DOI:** 10.3390/plants10061098

**Published:** 2021-05-30

**Authors:** Sahil Mehta, Amrita Chakraborty, Amit Roy, Indrakant K. Singh, Archana Singh

**Affiliations:** 1 International Centre for Genetic Engineering and Biotechnology, Aruna Asaf Ali Marg, New Delhi 110067, India; sahilmehtasm21@gmail.com; 2 EVA4.0 Unit, Faculty of Forestry and Wood Sciences, Czech University of Life Sciences Kamýcká 129, Suchdol, 165 21 Prague 6, Czech Republic; chakraborty@fld.czu.cz (A.C.); Roy@fld.czu.cz (A.R.); 3 Excelentní Tým pro Mitigaci (ETM), Faculty of Forestry and Wood Sciences, Czech University of Life Sciences Kamýcká 129, Suchdol, 165 21 Prague 6, Czech Republic; 4 Molecular Biology Research Lab, Department of Zoology, Deshbandhu College, University of Delhi, Kalkaji, New Delhi 110019, India; 5 Department of Botany, Hansraj College, University of Delhi, New Delhi 110007, India

**Keywords:** plants, microbes, pathogens, lipids, oxylipins, phosphatidic acid

## Abstract

Plant diseases pose a substantial threat to food availability, accessibility, and security as they account for economic losses of nearly $300 billion on a global scale. Although various strategies exist to reduce the impact of diseases, they can introduce harmful chemicals to the food chain and have an impact on the environment. Therefore, it is necessary to understand and exploit the plants’ immune systems to control the spread of pathogens and enable sustainable agriculture. Recently, growing pieces of evidence suggest a functional myriad of lipids to be involved in providing structural integrity, intracellular and extracellular signal transduction mediators to substantial cross-kingdom cell signaling at the host–pathogen interface. Furthermore, some pathogens recognize or exchange plant lipid-derived signals to identify an appropriate host or development, whereas others activate defense-related gene expression. Typically, the membrane serves as a reservoir of lipids. The set of lipids involved in plant–pathogen interaction includes fatty acids, oxylipins, phospholipids, glycolipids, glycerolipids, sphingolipids, and sterols. Overall, lipid signals influence plant–pathogen interactions at various levels ranging from the communication of virulence factors to the activation and implementation of host plant immune defenses. The current review aims to summarize the progress made in recent years regarding the involvement of lipids in plant–pathogen interaction and their crucial role in signal transduction.

## 1. Introduction

Plants are continuously exposed to “stress conditions” throughout their life cycle, starting from seed germination to the seed setting stage [1,2,3]. To progress and complete their life cycle even during single or combinatorial non-biological and biological stresses, plants adapt to continue thriving by evolving sophisticated defense mechanisms [4,5,6,7]. With multiple reports highlighting an increase in the frequency and incidences of these stresses in the past few decades [8,9], the most severe challenge at present is understanding the responses as well as adjustments that occur during the aversion of stress-triggered alterations in detail [3,10,11]. Most importantly, amongst many stresses, phytopathogens-incited biological stress affects the growth, development, and yield of various crop plant species such as wheat, maize, rice, barley, sugarcane, chickpea, pearl millet, cotton, lentil, faba bean, etc. Over time, upon being challenged by devastating phytopathogens, plants have evolved both constitutive and inducible mechanisms to defend themselves in the best possible manner. One such primary response is to change their growth rate and pattern to reduce the pathogenicity, which eventually modifies the host–pathogen interaction [12,13].

Several studies have revealed the role of lipids and lipid-related metabolites during plant–pathogen interactions over the past 25 years (Table 1). This role covers the lipoxygenase pathway-based production of defense-related oxylipins [14,15], the unsaturated fatty acid pathway for remodeling lipid composition in cellular and intracellular membranes [16], and the very-long-chain fatty acids (VLCFAs) pathway. In both eukaryotic and prokaryotic organisms (plants and phytopathogens), cellular membranes are rich in lipids—a primarily known class of hydrophobic molecules. As major constituents of both the plasma membrane and intracellular membranes, lipids play significant roles, such as being structural components of biological membranes, membrane fluidity, flexibility, and being involved in the regulation of membrane trafficking, cellular integrity, organelle identity, dynamic components of the enzyme system, the mediator in photosynthetic proteins, and signal regulation during cell metabolism and energy storage [17,18,19,20,21].

The mining of “omics-driven data” has revealed that plants harbor a diverse range of multiple complex and simple lipids such as fatty acids, galactolipids, sphingolipids, phospholipids, sulfolipids (SL), steroids, and waxes. However, plant membranes are typically represented by sphingolipids (glycosyl inositol phosphoceramides) (GIPC), ceramides (Cer), glucosylceramides (Gcer), glycerolipids, phospholipids (PL), SL, triacylglycerols (TAG), and galactolipids (GL), and sterols (free sterols, acylated sterol glycosides, steryl glycosides, and steryl esters) [22,23,24].

**Figure 1:**
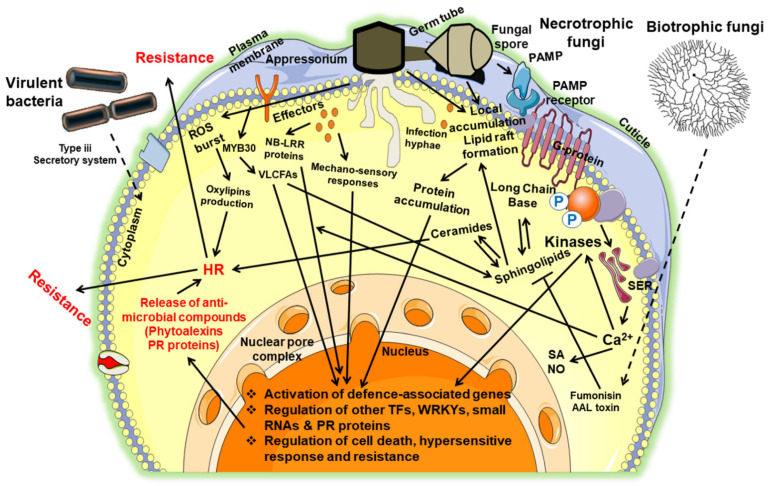
The schematic diagram represents the response of diverse lipids in plant–pathogenic interactions for various phytopathogens (bacteria and fungi). A variety of lipids and their associated proteins play an essential role in regulatory pathways of resistance against devastating pathogens. During pathogen infection, the downstream signaling pathways, including jasmonic acid-dependent and hypersensitive responses and the release of antimicrobial compounds, are modulated by ceramides (fungi), sphingolipids (fungi), VLCFAs (bacteria), MYB30 (bacteria), phospholipases (bacteria and fungi), and Phyto-oxylipins (fungi). At the back end, this mechanism of imparting resistance against phytopathogens is directly or indirectly controlled by ROS bursting, calcium signaling, mechano-sensory responses, lipid raft, and the surface perception of elicitors by interacting with other transcription factors, phospholipases, and kinases. Additionally, during pathogen infection, the “cuticle” (made up of certain cutin monomers or wax components) responds faster to the pathogen elicitors. Thus, it activates plant disease resistance through PTI and ETI.

Due to the recent advancements in omics technology and sensitive instrumentation, including gas chromatography, mass-spectrometry, and high-pressure liquid chromatography, lipids and their associated functionality have been identified to play a central role during plant–pathogenic interactions [25,26,27,28,29]. These interactions occur at multiple stages and are regulated by lipids derived from both hosts and microbes. Furthermore, lipids impact pathogen development, host–microbe communication, and antimicrobial activities, facilitate the signaling of proteins to cellular membranes, and function as signaling molecules (Figure 1). Additionally, many lipids derived from a host or pathogen have been identified as an innate signal that elicits compounds by the primary defense response ([30]; Table 1 and Table 2). Besides the physical impediments on the epidermal cell surface, several other components such as waxes, terpenoids, cutin, fatty acids, phenolics, aldehydes, and polysaccharides are distributed abundantly and help to resist pathogenic attack [31,32,33]. With the implementation of “span genomics”, many resistance gene analogs (RGAs) or R-gene repertoires have been identified, and some of these R genes may function via lipid-mediated pathways [34,35,36]. Overall, lipid signals influence plant–pathogen interactions to a great extent. This article highlights the involvement of different classes of lipids in defense signaling during plant–pathogen interaction.

**Table 1:** Involvement of membranous lipids and their associated enzymes during plant–pathogen interaction.

Substrate	Enzyme Involved	Products	Functions	References
Glycerophospholipids	Phospholipase A	Lysophopholipids, free fatty acids	Resistance against *Xanthomonas* species; induction of auxin signaling; elicitation in the biosynthesis of phytoalexin; induction of pathogen-associated molecular protein (PAMP)-triggered immunity and effector-triggered immunity (ETI) against pathogens	[37]
Phosphorylcholine, Phosphorylethanolamine	Phospholipase D	Phosphatidic acid	Control of the signaling of phosphatidic acid; resistance against drought, salt, and cold stress; inhibiting spore penetration and providing fungal resistance; induction of effector-triggered immunity and PAMP-triggered immunity against pathogens; regulating the signaling of Jasmonic acid and salicylic acid; wounding response in plants	[34,38]
Phosphatidylinositol-4,5-biphosphate, Phosphatidylinositol-4-phosphate	Phosphoinositide-specific phospholipase C	Phosphatidic acid, Diacylglycerol, and Inositol phosphate	Plant protein localization induces effector-triggered immunity and PAMP-triggered immunity during pathogen attack; resistance during drought, heat, and salt stress	[39,40]
Phosphorylcholine, Phosphorylethanolamine	Non-specific phospholipase C	Phosphorylalcohol, Diacylglycerol	Root development; response during cold and salt stress	[41]
Diacylglycerol	Diacylglycerol kinase	Phosphatidic acid	Effector-triggered response against pathogens; signaling of defense response during salt and cold stress; enhances plant growth and development	[42]
Phosphatidic acid	Phosphatidic acid kinase	Diacylglycerol pyrophosphate	Induction of an ABA-mediated response to pathogen attack	[43,44]
Phosphorylated Phosphatidylinositol	Phosphatidylinositol kinase	Phosphoinositides	Induction of a phosphatidylinositol-mediated stress response	[45]
Phosphatidyl ethanolamines	Fatty acid amide hydrolase	*N*-Acylethanolamines	Affects abscisic acid signaling and provides resistance against pathogens	[46]
Ceramide	Sphinganine *N*-acyltransferase	Sphingolipids	Rescues plants from the lethal effects of mycotoxins; modulates cell death processes during pathogen attacks	[47]
Diacylglycerol	Galactolipases	Galactolipids	Precursor of Jasmonic acid synthesis in wounded leaves; activates R gene-mediated signaling	[48]
Polyunsaturated fatty acid	Nonenzymatic free-radicalmechanism	Phytoprostane	Induces the expression of glutathione S-transferases and glycosyltransferases; enhances the metabolism of phytoalexins; responds in oxidative stress	[26,49]
β-sitosterol	Brassinosteroid-6-oxidase 1	Brassinosteroids	Resistance against bacterial blight disease and fungal pathogen	[50]
Linolenic acid	Jasmonic acid carboxyl methyltransferase	Jasmonates	Induces defense-related genes; resistance to *B. cinerea* attack	[51,52]

## 2. Primary Response of Host Plant against Microbial Infection

The cuticle can be regarded as a storehouse of signal translators when a pathogen finds a susceptible and ideal host to infect and colonize. Cuticular lipids are present on cell surfaces and act as messenger molecules during plant–pathogen interaction. The signal perception of such events depends upon the metabolism of lipid messengers such as oxylipins and phospholipases (Table 1). Several transcription factors regulate and initiate the lipid pathways involved in defense and eventually lead to the death of infected cells [53,54,55]; for example, *MYB30* (a Myb-domain transcription factor), a transcription factor that exhibits rapid, specific, and transient transcriptional initiation in response to *Xanthomonas campestris* infection and acts as a positive regulator of hypersensitive cell death in *Arabidopsis* [56].

Similarly, the overexpression of *MYB30* in tobacco and *Arabidopsis* has been shown to contribute to the highly resistant nature of transgenic *Arabidopsis* and tobacco to powerful fungal pathogens. It is worth noting that a total of 14 lipid-associated genes are activated at an early response during *X. campestris*-infection. However, the upregulation of cuticular genes, VLCFAs, and their derivatives, particularly through *MYB30*, might be responsible for lipid signaling related to hypersensitive cell death response [56].

In *Arabidopsis att1* (aberrant induction of type III genes) mutant, the *Pseudomonas syrinage* infection resulted in 70% reduced cutin content [57]. *ATT1* encodes for CYP86A2, a cytochrome P450 monooxygenase that catalyzes fatty acid oxidation and the biosynthesis of extracellular lipids such as cutin. In *att1*, CYP86A2 is not functional; therefore, a decrease in cutin content was observed. Eventually, this facilitates the expression of bacterial type III gene *AvrPto* and *HrpL* as well as enhances the severity of disease caused by *P. syringae*.

In another report, it was observed that cutin monomers promote appressorium formation and spore germination in two devastating pathogens: *Magnaporthe grisea* and *Colletotrichum gloeosporioides*. In contrast, cutin monomers stimulate the formation of an appressorial tube in *Blumeria graminis* [31]. Thus, *CUT2*, a cutinase encoding gene, is involved in the penetration peg formation of *M. grisea* fungus. *CUT2* has a dual role as a barrier to pathogens and a signaling regulator during microbial pathogenicity and plant defense. In another study, cutinases were observed to be secreted from a powdery mildew-causing pathogen, *Erysiphe graminis*, and to induce appressorium formation on a host plant. However, removing such cuticular waxes altered cuticle thicknesses due to the symptoms of multiple avr genotypes 4 (*sma4*). The *Arabidopsis* mutants, *lacs* (Long-Chain Acyl-Coenzyme A Synthetase) and *bre1* (E3 ubiquitin ligase), suppress spore germination and comparatively reduce the conidial growth of *B. graminis* on barley. Thus, the reduced permeability and thickness of the cuticle arrest the surface invasion and restrict the entry of pathogens [31,32,58]. In addition, cuticles also differentiate the significant germination processes of various fungi and regulate the plant–pathogen infection process. For example, in the *Arabidopsis* *sma4* mutant, the avirulent strain of *P. syringae* pv. *tomato* can easily cause infection, and this mutant exerts normal susceptibility towards one of the most devastating biotrophic pathogens, *Erysiphe cichoracearum*, but not the necrotrophic fungus Botrytis cinerea. This is because of the *LACS2*-encoded cutin-based inhibition of spore germination and penetration [32,33,59].

Additionally, *Candidatus* Liberibacter spp., causing Huanglongbing disease, shows virulence via the secretion of lipopolysaccharides (LPS) that help in the colonization of citrus fruits [60]. Moreover, bacterial pathogens activate non-specific phospholipase C (NPCs) and activate PI-PLC during their infection period. Upon infection with *Ralstonia solanacearum* (Strain 8107), the activity of both phospholipase D (PLD) and phospholipase C (PLC) get decreased due to the silencing of the *Nb*SEC14 gene (the *Sec14*-protein superfamily codes for phosphatidylinositol/phosphatidylcholine transfer protein). Recently, in *Arabidopsis*, the expression of the *NPC6* gene (*Non-specific phospholipase* *C6*) has been observed to be downregulated after treatment with *Phytophthora* *parasitica* and flg22 (Flagellin 22). However, *NPC3* (*Non-specific phospholipase* *C3*) and *NPC4* (*Non-specific phospholipase* *C4*) have been observed to be activated in response to *Golovinomyces orotii*, *B. cinerea*, *P. parasitica*, and *P. syringae* treatment. Furthermore, *NPCQ* (*NPC Intracellular Cholesterol Transporter 1*) and *NPC4* expression were responsive to two other bacterial elicitors named HrpzZ and flg22 [61].

**Table 2:** Molecular regulation and response strategies of plants upon successful invasion by pathogens.

Crop	Genes	Pathogen	Lipase Involved	Response(s)	References
Tomato	*PI-PLC* (Phosphatidylinositol-specific phospholipase C), *Sl*PLC4, *Sl*PLC6	*Cladosporium fulvum*	Lipase 3	Pi-PLC signaling induces hypersensitive response and resistance against pathogens	[30,62]
Rice	*Os*SBP (*Selenium-binding protein homolog*)	*Xanthomonas oryzae*	Extracellular lipase	Enhances the production of phytoalexins in plant defense	[63]
Pepper	*Bs1 (Resistance to Bacterial Leaf Spot*	*Xanthomonas campestris*	GDSL-type lipase	Induction of other defense genes but inhibition of the expression of *Ca*WRKY1	[60,64]
Brassica	*Xca4* (*Controlled resistance to X. campestris race 4*)	*X. campestris*	Extracellular lipase	Upregulation of ROS scavenging enzymes	[36,65]
Flax	AvrP4, AvrM, AvrL567	*Melampsora lini*	Lipase 8	Triggers hypersensitive response	[66]
Brassica	*BLMR1* *(Blackleg resistance protein variant 1)*	*Clostridium chauvoei*	GDSL-type lipases	Induction of plant defense signaling pathways	[65]
Soybean	Avr1b-1	*Phytophthora sojae*	*sn*-1,3 selective lipase	Induction of defense responses such as the accumulation of phenolic compounds, phytoalexins	[67]
Brassica	*Bn*IGMT5.a (*Indole glucosinolate o-methyltransferase 5*)	*P. sojae*	GDSL-type lipases	PAMP-triggered immunity response	[65]
Arabidopsis	*ATR13, ATR1* (*RxLR* effector encoding gene)	*Hyaloperonospora parasitica*	Pathogenesis-related lipases	Cognate resistance protein RPP13^Nd^	[67,68]
Potato	Avr3	*Phytophthora infestans*	--	Encodes proteins with elicitor functions	[69]
Squash	Viral coat protein	*Cucumber Mosaic Virus*	--	Modulates the accumulation of 2b protein	[70]
Papaya	Viral coat protein	*Papaya Ring Spot Virus*	*Carica papaya* lipase	Mediates RNA-mediated natural defense	[70]
Rice	*Xa21* (*Xanthomonas oryzae* pv. *oryzae* resistance *21*)	*X. oryzae*	Lipase A	Encodes a receptor-like kinase as well as binding to WRKY transcription factor	[60,71]
Rice	Chitinase	*Magnaporthe oryzae*	Lipase A	Activates expression of defense responsive gene	[72]

## 3. Lipids and Lipid-Derivatives Involved in Host Signaling Response

The plant oxylipins or Phyto-oxylipins (POs) are a class of oxidized lipids produced in a wide range of stressed conditions that further induce stress-activated signaling pathways. They are found as esterified glycerolipids or in a free form. The PO signatures are plant-specific and regulated by the kind of pathogens, affected plant organs, and the pathogen’s lifestyle [73,74]. For example, tobacco plants have been observed to accumulate α-dioxygenase (α-DOX) and **9**-lipoxygenase (LOX) products after infection with *P. syringae*. In contrast, potato and tomato plants accumulate 9(S)- and 13(S)-polyunsaturated fatty acid hydroperoxides upon infection by *P. infestans* and *B. cinerea*, respectively [75,76,77,78].

Within the huge variety of POs, jasmonic acid (JA) and its derivatives are well-known LOX-derived molecules that are quickly accumulated in pathogen-damaged tissues/plants [52,79]. Within the class of JA-derivatives, the methyl-JAs are volatile compounds produced for signaling communication in an intra and interspecific manner. According to the reports related to pathogen response, (+)-7-iso-jasmonoyl-L-isoleucine (JA-Ile) has been observed to be accumulated within five minutes in the leaves of *Arabidopsis thaliana* distantly located from the wounding areas [68,80]. Furthermore, in this regard, the trio signaling process of Ca^2+^, reactive oxygen species (ROS), and electrical coupling-mediated plasma membrane potential (*V*m) regulate long-distance signaling for a quick response instead of direct hormonal movements [81,82]. Furthermore, JA induces signaling that encourages the pump or ion channel encoding genes and favors the release of long-distance Ca^2+^-signaling [83,84]. However, at present, complete knowledge of pattern-associated molecular patterning and damage-associated molecular patterns in PO-mediated signaling during pathogen attack is lacking. The PLs are the integral components of the membrane bilayer and provide the perceiving areas during plant–pathogen interaction. They have regulatory roles in plant immunity. PL-based signaling includes the activation of phospholipases (PLs) and protein kinases (PKs) to induce a variety of intracellular signaling molecules by phosphorylating or cleaving the bonds of PL to regulate diverse physiological processes of plants [85,86]. There are three categories of PLs based on their substrate specificities and enzymatic activities: Phospholipase A (PLA), PLC, and PLD.

PLA triggers the hydrolysis of acyl parts from the targets at *sn*-1 and *sn*-2 positions of glycerol derivatives. PLAs target monogalactosyldiacylglycerol (MGDG), phosphatidylethanolamine (PE), digalactosyldiacylglycerol (DGDG), phosphatidylcholine (PC), and triacylglycerol (TAG) by inducing hydrolysis. Interestingly, both PLC and PLD induce the cleavage of phosphodiester bonds of PL that differ in their proximity and terminal positions [87,88]. There are two kinds of PLC in plants: PC-specific PLCs and phosphoinositide (PI)-specific PLCs. PI-PLCs trigger the cleavage of phosphatidylinositol-4-phosphate [PtdIns (4) P], phosphatidylinositol-4, 5-bisphosphate [PtdIns (4,5) P2], and phosphatidylinositol, while PC-specific PLCs target phosphatidylserine (PS), PC and PE [39,89,90]. PLDs have a wider range of substrates, such as PC, PE, PI, PS, N-acyl phosphatidylethanolamine, and phosphatidylglycerol (PG). Additionally, PI5K, PI4K, PI3K, PI4P 5-kinase, PI3P 5-kinase, and PI5P 4-kinases are the kinases that act on PI by phosphorylating the inositol ring on the D5, D4, and D3 positions [91].

Phosphatidic acid (PA) is known to be central player for lipid transport across membranes in addition to its defensive role, and it also acts as a precursor for PI, PC, and PE [92]. Furthermore, PAs play a crucial role in stomatal closure with the aid of ABA-mediated signaling by targeting the activities of the respiratory burst oxidase homologs (*RBOHD* and *RBOHF*) [43,93]. Phospholipase D α1 (PLDα1) generates PA, which binds with *RBOHD* and *RBOHF* and triggers ROS production. This ROS production stimulates stomatal closure. The role of PLDα1 and *RBOH* in stomatal closure is also supported by the observation that the mutants of PLDα1 and *RBOH* show insensitivity to ABA.

On the other hand, PA also binds with the ABI protein phosphatase 2C (the negative regulator of ABA-mediated signaling) to inhibit phosphatase activity [94,95]. It has also been suggested that PA can act as a ubiquitous signaling component for diverse abiotic and biotic stress-induced signaling pathways. This is because of the differential binding of PA to target enzymes or proteins that alter their localization and activity. The mutation in *At*PLDβ1 (*Phospholipase D* β1-encoding gene) shows compromised resistance against *B. cinerea*, elucidating the positive role of PA in the JA pathway.

**Figure 2:**
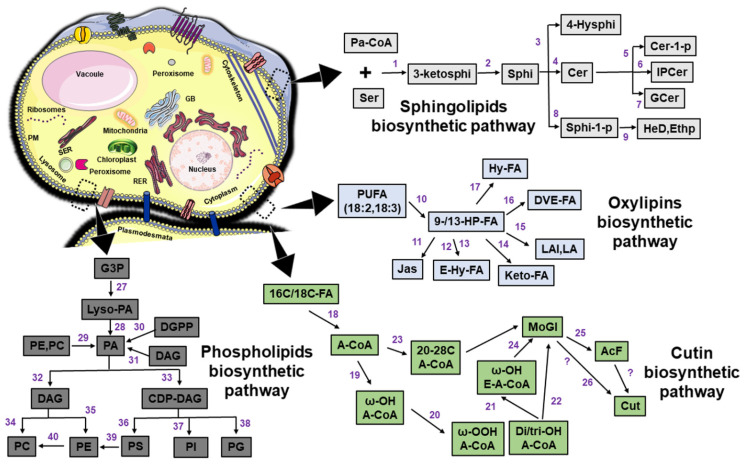
Schematic representation of various lipid-associated biosynthetic pathways in plants. The abbreviations used in figures are as follows: Pa-CoA: Palmitoyl-CoA, Ser: Serine, 3-ketosphi: 3-ketosphinganine, Sphi: Sphinganine, 4-Hysphi: 4-Hydroxysphinganine, Cer: Ceramide, Cer-1-p: Ceramide-1-phosphate, IPCer: Inositol-phosphorylceramide, Gcer: Glucosylceramide, Sphi-1-p: Sphinganine-1-phosphate, HeD: Hexadecanal, Ethp: Ethanolamine phosphate, PUFA (18:2,18:3): Polyunsaturated fatty acids, 9-/13-HP-FA: 9- or 13-hydroperoxides fatty acids, Jas: Jasmonates, E-Hy-FA: Epoxy-hydroxy fatty acids, Keto-FA: Keto-fatty acids, LAl: Leaf aldehydes, LA: Leaf alcohols, DVE-FA: Divinylether fatty acids, Hy-FA: Hydroxy fatty acids, 16C/18C-FA: 16- and 18-carbon fatty acids, A-CoA: Acyl-Coenzyme A, ω-OH A-CoA: Acyl-CoA product bearing a terminal hydroxy group at ω-position, Di/tri-OH A-CoA: Di/tri oxidated acyl-CoA product, ω-OOH A-CoA: Acyl-CoA product bearing a terminal peroxide group at ω-position, ω-OH E-A-CoA: Expoxy acyl-CoA product bearing a terminal hydroxy group at ω-position, 20-28C A-CoA: 20-28 carbon acyl-CoA, MoGl: Monoacylglycerols, AcF: Acyl ferulate, Cut: Cutin, G3P: Glycerol-3-phosphate, Lyso-PA: 1-oleoyl-2-hydroxy-*sn*-glycero-3-phosphate, PA: Phosphatidic acid, PE: Phosphorylethanolamine, PC: Phosphorylcholine, DGPP: Diacylglycerol pyrophosphate, DAG: Diacylglycerol, CDP-DAG: Cytidine 5’-diphosphate diacylglycerol, PG: Phosphatidylglycerol, PI: Phosphoinositide and PS: phosphoserine. In here, the enzymes related to biosynthetic machinery are shown in purple-colored numbers: 1: Serine palmitoyltransferase, 2: **3**-ketosphinganine reductase, **3**- Sphinganine hydroxylase, 4: Sphinganine N-acyltransferase, 5: Ceramide kinase, 6: inositol-phosphorylceramide synthase, 7: glucosyl-ceramide synthase, 8: Sphinganine kinase, 9: sphinganine phosphate lyase, 10: Lipoxygenase, 11: Allene oxide synthase, 12: Epoxy-alcohol synthase, 13: Peroxygenase, 14: Lipoxygenase, 15: Hydroperoxide lyase, 16: Divinylether synthase, 17: Hydroperoxide synthase, 18: Long-chain acyl-CoA synthetase (LACS) proteins, 19: CYP86A (Cytochrome P450, Family 86, Subfamily A, Polypeptide), 20: CYP77A6 (Cytochrome P450, Family 77, Subfamily A, Polypeptide 6), CYP86A2 (Cytochrome P450, Family 86, Subfamily A, Polypeptide 2), 21: CYP77A4 (Cytochrome P450, Family 77, Subfamily A, Polypeptide 2), 22: Glycerol-3-phosphate acyltransferase (GPAT), 23: Fatty acid elongase complex (FAE), 24: Glycerol-**3**-phosphate acyltransferase (GPAT), 25: *Deficient In Cutin Ferulate* (DCF), 26: Cutin synthase (CUS), ?: Unknown, 27: G3P acyltransferase, 28: Lyso-acyltransferase, 29: Phospholipase D, 30: DGPP phosphatase, 31: Diacylglycerol kinase, 32: Phospholipase C, 33: Cytidine **5**’-diphosphate diacylglycerol synthase (CDS), 34: Cholinephosphotransferase, 35: Phosphoethanolamine cytidylyltransferase, 36: Phosphoserine synthase, 37: Phosphoinositide synthase, 38: PG phosphate synthase, PGP phosphatase, 39: Phosphoserine decarboxylase and 40: Phosphatidylethanolamine N-methyltransferase.

Lysophospholipids (LPL) and PI also have a role in plant defense (Figure 1). PI is produced by phosphatases and lipid kinases (Figure 2), as described earlier. They serve as a precursor for stress-signaling lipids such as DAG and inositol phosphatases [96]. The signaling pathway of LPL is involved in an overlapped manner with PA-induced signaling [97,98]. The activity of PLAs induces LPL generation from the glycerophospholipids such as sphingosylphosphorylcholine and sphingosine-1-phosphate lysophosphatidylcholine. The specificity and the signaling activity are defined by the acyl chain position, length, saturation degree, and phosphate head group. LPL-specific lipid signaling is involved in pest attack, pathogen infection, and wounding [45,99].

In plants, sphingolipids are the major signaling structural components of plasma membranes as well as other endo-membranes and play crucial roles in the host–pathogen interactions and other stress responses [47,100]. Chemically, sphingolipids are ceramide-containing fatty acid nonglycerol lipids connected to long-chain amino alcohol [101,102]. Because of their highly hydroxylated nature, sphingolipids improve membrane properties such as stability and permeability and even regulate protection against fungal pathogens and other environmental stresses [102]. This can be understood by the fact that sphingolipids are used to induce cell death in plants, which eventually limits the pathogen spread from damaged tissues. For example, a sphingosine analog is produced by *Alternaria alternata* f.sp. *lycopersici*, which serves as a virulence factor and causes programmed cell death both in plants and animals [103].

Additionally, the AAL toxin and fumonisin (toxins of *Fusarium moniliforme*) impede the biosynthesis of sphingolipids by targeting ceramide synthase, resulting in the accumulation of long-chain bases. Eventually, this increased level of long-chain bases or their ratio to ceramide signals causes pathogen-based programmed cell death [104,105]. The R protein *ASC1* recognizes these toxins and confers resistance by mediating alternative ceramide synthesis and the transfer of GPI-anchored protein to the Golgi apparatus from the endoplasmic reticulum. Thus, the transfer of ceramides to the membrane eventually plays an essential role in modulating programmed cell death.

## 4. Phospholipase and Its Role in Defense Signaling

**Figure 3:**
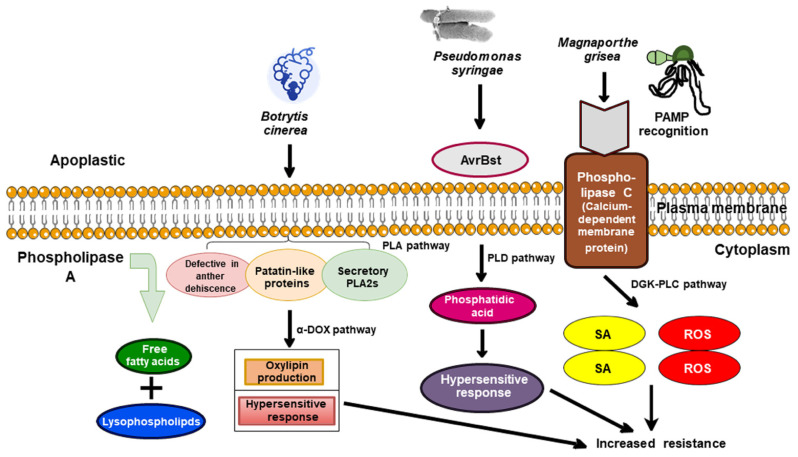
Surface signaling responses of hosts after the primary invasion of pathogenic species. During an immune response, the fundamental tenet is the ability to detect the presence of devastating agents (such as phytopathogens) followed by activating the defense responses. In plants, immunity is governed by transmembrane pattern recognition receptors (PRRs) and other downstream cellular components. These downstream immunity-related components include key enzymes—phospholipases—which draw much of our attention. PLAs are a superfamily of functionally diverse enzymes that actually govern membrane dynamics. The large superfamily of Phospholipases is divided into three sub-families: Phospholipase A (PLA), Phospholipase C (PLC), and Phospholipase D (PLD). These Phospholipases often hydrolyze various plasma membrane and intracellular membranes-derived phospholipids, phosphatidylinositol, and related-derivatives to generate signaling molecules such as phosphatidic acid, oxylipins, free fatty acids, and lysophospholipids as well as other molecules (inositol trisphosphate, diacylglycerol) that ultimately impart resistance against bacteria (*Pseudomonas syringae*) as well as fungi (*Botrytis cinerea* and *Magnaporthe grisea*).

Phospholipase plays a versatile role in pathogen response and acts rapidly on the perception of stimuli [30]. The transcription of PLA, PLD, PLC, and diacylglycerol kinase (DGK)-encoding genes and their enzymatic activities have been reported to be enhanced upon elicitor treatment and pathogen infection in tomato (*Solanum lycopersicum*), rice (*Oryza sativa*), Arabidopsis (*A. thaliana*), and tobacco (*Nicotiana tabacum*) [106,107,108] (Table 2). Pathogen recognition initiates the cascade of phospholipase-dependent signaling pathways. PLAs hydrolyze membranous PL to produce LPL (Figure 2) and free fatty acids (FFA). PLAs are categorized into DAD (*defective in the anther dehiscence*), patatin-like proteins (pPLAs), and secretory phospholipase A2 (PLA2s). However, SAG101 (*senescence-associated carboxylesterase 101*), PAD4 (*phytoalexin deficient 4*), and EDS1 (*enhanced disease susceptibility1*) are the main components that shuttle during signaling via the activities of PLAs [30]. In a study, perforin-like proteins (PLP) and patatin-related phospholipase 2A (pPLA 2α) are activated after the invasion of *P. syringae* pv. *tomato* or *B. cinerea* [30]. In *Arabidopsis*, the modified regulation of PLP2 alters plant susceptibility to *P. syringae* pv. *tomato* or *B. cinerea*, whereas the expression of PLP2 promotes the growth of necrotizing pathogens but enhances the resistance towards devastating viral pathogen *CMV* (Figure 3). PLP2 expression promotes oxylipin accumulation in advanced stages of *Botrytis* invasion via α-DOX pathways and restricts the spreading of hypersensitive response (HR) [30]. Conclusively, PLP2 activities may be driven by pathogens to accelerate colonization within the host. In *Arabidopsis*, PLD and PLA activities are supposed to be interlinked during the plant defense response. When *P. syringae* expressing AvrBst (an effector molecule) infects the Pi-0 ecotype of *Arabidopsis* (Figure 3), PA production occurs through PLD pathways, which is mandatory for HR responses. A loss of function mutation in *SOBERI1* (*SUPPRESSOR OF AVRBST-ELICITED RESISTANCE 1*) (exhibiting PLA activities) displays the resistance phenotype in the Pi-0 plant. In contrast, in the Col-0 a-susceptible *Arabidopsis* ecotype, *SOBERI* (PLA2) activity competes for the substrate with PLD, thus reducing the accumulation of PA to elicit an AvrBst response [109].

Secondly, PLC (Ca^2+^-dependent membrane protein) responds to pathogen elicitors after the invasion at the membrane region and triggers the PLC pathway through PAMP recognition [30]. In rice, *Os*PLC1 (Phospholipase C1) gene expression was induced during the incompatibility between *M. grisea–O. sativa* interaction, which confirmed the role of *Os*PLC1 in signaling during pathogen infection [30]. In the *S. lycopersicum* cell suspension culture, the upregulation of *Cf-4* (a membrane-anchored protein that provides resistance to *Cladosporium fulvum*) resistance gene via the activity of Avr4 (a cognate pathogen effector) induced a rapid accumulation of PA through the DGK-PLC pathways [110]. Further, *Slplc4* gene silencing impaired Avr4/*Cf4*-stimulated HR and facilitated the susceptibility of *Cf-4* plants to *Cladosporium fulvum* [62]. However, *Sl*PLC6 silencing in tomato did not influence Avr4/*Cf-4* induced HR but compensated resistance induced by R-genes such as *Prf/Pto* (resistance to *Phytophthora infestans*/*Pseudomonas syringae* pv. *tomato*), *Ve1* (an encoding protein for resistance to *Verticillium* wilt disease), and *Cf-4* [111]. Additionally, the abrogation of PA through n-butanol compensates for cell wall-based resistance in *Arabidopsis* to sensitive powdery mildew pathogen and AvrRpm1/RPM1 triggered immunity. This disease-resistant protein, RPM1, is recognized as the AvrRpm1 type III effector avirulence protein from *Pseudomonas syringae* [111]. The molecular dissection of *Arabidopsis* defense response revealed that *At*PLDδ contributes to invasion resistance toward sensitive mildew pathogens.

Interestingly, several lipid-signaling genes have also been observed to induce resistance; for example, *At*PLDβ1 (which enhances the concentration of oxidative species, salicylic acid, and increments the resistance to *P. syringae*), *Os*PLDβ1 (elevates the resistance level to diverse pathogens in rice), and *Sl*PLD β1 (enhances resistance in host species) [108,112]. Indeed, *EDS1* or PAMP-dependent host response tightly controls negative regulators such as *PUB13* (an E3-ubiquitin ligase containing a U-box domain), MAPKKK (mitogen-activated protein kinase kinase kinase), *EDR1* (*enhanced disease resistance 1*), and Ca^2+^/SR1 (calmodulin-binding transcription factor). Interestingly, PI4KIIIβ1 and PI4KIII2β regulate *FLS2* (encodes for LRR receptor-like serine/threonine-protein kinase) homeostasis, and flagellin recognized PRR, and negatively regulate salicylic acid (SA) signaling to suggest a supposed braking mechanism for pattern-triggered immunity (PTI) [108,113].

Fungal pathogens may alter their growth activities and morphology in response to the surface signals of a host. When *Ustilago maydis* infects maize plants, it converts the non-filamentous pathogenic form into a filamentous form to enhance invasion and colonization. The lipase activities of the fungus *U. maydis* are solely responsible for the consequential liberation of lipids on a surface that helps to change its pathogenic nature [114].

Additionally, some lipases play a primary role in pathogenicity in the panicle blast disease of rice caused by *Burkholderia glumae*. The pathogenic strain of *B. glumae* (AU6208) shows the secretion of Lipase A (LipA) on rice that is regulated by tof1/R (a quorum-sensing system) [115]. The tof1/R in *B. glumae* is a specific signaling system that depends upon the cell density produced by the *tof1* (topoisomerase I-interacting factor) gene, whereas *tof1* regulators attach with signals at a threshold amount and mediate the desired gene expression. However, the *B. glumae* AU6208 strain with the *tof1* mutant shows a slight concentration of lipase but does not release any N acyl-homoserine lactone (AHL) compounds. It is more likely that tof1/R-mediated LipA regulation is more common at the transcriptional level than the secreted protein level, as depicted by a study on a mutant (*B. glumae* AU6208 *tof1*). The virulence assessment of mutant and LipA regulation by the tof1/R system suggested the significance of lipase during infection that positively mediates bacterial populations. The above findings summarize the activity of *B. glumae* lipase and its interaction with the host lipid that may trigger plant responses [115,116]. Moreover, the plant develops several molecular regulation and response strategies associated with lipase activities after the successful infection of pathogens (Table 2).

## 5. Jasmonate-Derived Oxylipins as the Critical Mediator in Plant–Pathogen Interaction

Oxylipins are a diverse class of oxidized lipids that are abundant in both plants and phytopathogens [117]. These are distributed as free forms, esterified with glycolipids/PL, or attached with other compounds, including isoleucine and methyl groups. Oxylipins can be derived from the free radical-catalyzed oxidation of polyunsaturated fatty acids (PUFA) via enzymatic and non-enzymatic pathways. Among these, an increased concentration of phytoprostane is observed in plants after successful exposure to pathogens that cause oxidative stress [118]. The application of phytoprostane to *Arabidopsis* cells restricts the cell death elicited by toxins and detoxifying enzymes (glycosyltransferase and glutathione S-transferases), the activation of mitogen-activated protein kinases (MAPK), and the biosynthesis of phytoalexins [118]. *LOX*s gene expression is induced in diseased plants and associated with resistance [117]. The expression level of two LOX—9-LOXs and 13-LOXs—is significantly enhanced during pathogen attack, and they induce signaling cascades of the defense response [119].

### Successful Chase of Jasmonate: Elucidates Defense Clues during Pathogen Attack

Classically, jasmonate is a typical oxylipin that has an important role in the defense response during a pathogenic attack and it induces antimicrobial activities [117,120]. Mostly, pathogens such as *X. campestris* pv. *phormiicola*, *Streptomyces scabies*, and *Pseudomonas cannabina* pv. *alisalensis* show an enhanced production of 2,4-diamino-1,5-diphenyl-3-hydroxypentane (COR) compounds [121,122]. Oomycetes and fungal pathogens secrete protein-rich effectors that activate JA signaling in *Arabidopsis*, along with susceptibility to the effector protein SECRETED IN XYLEM (SIX). The F0 causes the expression of *LOB DOMAIN CONTAINING PROTEIN 20* (*LBD20*), which regulates the downstream function of *MYC2* (encodes an MYC-related transcriptional activator that binds to an extended G-Box promoter motif and interacts with Jasmonate ZIM-domain proteins) and *COI1* (encoding coronatine-insensitive protein 1, which plays a role in JA-regulated defense processes and coronatine-like elicitors perception) and increases pathogenesis [123].

*LBD20* plays a crucial role in the JA-mediated defense response, and its expression causes the inhibition of JA signaling marked by *VSP2* (Vegetative storage protein 2) and *THI2.1* expression. Nevertheless, no effect of *LBD20* has been observed on the *PDF1.2* function. This indicates that *LBD20* may promote pathogenesis. Interestingly, this THIONIN 2.1 protein functions as a defense factor that exerts its toxic effect at the cell membrane level [123].

Additionally, the HARxL44 effector protein in *Hyaloperonospora arabidopsis* induces ET/JA signaling, suppresses the SA response, and increases host infection susceptibility through the interference of *MED19a* (mediator of RNA polymerase II transcription subunit 19a) [124]. HaRxL44 induces the proteasome-mediated degradation of MED19a and causes enhanced infection in host species.

In some cases, symbionts and pathogens also release protein effectors to inhibit JA- signaling. For example, the SSITL (*Sclerotinia sclerotiorum* integrin-like) protein suppresses ET/JA signaling pathways during primary infection [125]. Similarly, the MiSSP7 protein (MYCORRHIZA-induced SMALL SECRECTED PROTEIN 7) of *Laccaria bicolor* enters into *Populus tremuloides* through PI3P (phosphatidylinositol **3**-phosphate)-regulated endocytosis associated with *Pt*JAZ6, restricts *Pt*JAZ6 degradation in the host nucleus via SA signaling regulation, and promotes symbiosis [126]. Comparatively, SP7 (SECRETED PROTEIN 7) secreted by *Glomus intraradices* interacts with the host transcription factor ERF19 in the nucleus and inhibits the ET-derived plant defense, elevating *G. intraradices*–*Medicago truncatula* symbiosis [127].

The pathogens enhance their activities from the surface level to the nucleus using different lipases and cause pathogenesis (Table 3). For example, during the invasion of the *P. syringae* pv. *tomato* (Pst) strain, DC3000 releases phytoalexins such as coronatine that bind to *COI1* (JA-receptor COR insensitive 1) and mimic the structure of JA-IIe, leading to the activation of a JA-mediated response. COR or JA-IIe mediates the activation of JA2-like transcription factors that initiate *SAMT1* and *SAMT2* (S-Adenosylmethionine transporter 1/2) expression, which further regulates SA deactivation via methylation [128]. In *Arabidopsis*, activating the JA pathways that are regulated through E-2-hexenal enhances susceptibility to the Pst strain [129]. The characterization of *OPR3* (*Oxophytodienoate-Reductase 3*) mutant shows that 12-oxo-phytodienoic acid (OPDA) (a precursor for JA biosynthesis) upregulates both *COI1*-dependent and *COI1*-independent genes that do not respond to JA. In tomato, *P. syringae* (Pst strain) can overcome host defenses and colonize onto plant apoplastic regions and their adjoining cells. The secretion of coronamic acid (CMA) and coronafacic acid (CFA) from COR (coronatine) activities modify the CFA operon and promote the binding of CFA ligase attachment with CMA and CFA with an amide linkage [130]. The toxic compounds released by the bacteria bind to COI receptors that regulate JA-derived responses. The significant role of COR helps in stomatal opening and promotes the entry of bacteria into the leaf. Instead of COR synthesis, the ability of Pst depends upon the entry of effector proteins into the host through T3SS (type III secretion system). T3SS encoded by *hrc* (*Hypersensitive response and pathogenicity*) and *hrp* (*Hypersensitive response conserved*) genes and is used by bacteria to insert type III effector in a target host to suppress ETI and PTI. Primarily, Pst secretes approximately 35 effectors, such as mono-ADP-ribosyltransferases (hopu1 and hopf2), an E3 ligase (Avrptob), phosphothreonine lyase (*hopai1*), cysteine proteases (AvrPphb and AvrRpt2), etc. [131].

*OPR3*-silenced tomato (*Sl*OPR3) mutants exhibit an elevated susceptibility toward necrotrophic pathogen *B. cinerea* due to a decline in JA-IIe and OPDA levels [131]. Further, it has also been shown that only OPDA treatment could restrict *B. cinerea* resistance in *Sl*OPR3 transgenic plants by callose deposition. The inoculation of JA-deficient *opr7-opr8-2* and *GLV*-deficient mutant (green leaf volatile) indicates the role of JA and *Lipoxygenase 10* (LOX10) during bacterial/fungal infection [119,131].

## 6. Conclusions and Future Perspectives

The plant–pathogen interaction is a highly complex phenomenon. It includes a breadth of interrelated networks of signaling components and results either in a state of resistance or susceptibility. In this regard, lipid and lipid-derivatives act as pivotal modulators of interkingdom communication that include resistance, invasion, and pathogenesis mechanisms in plants. During recent years, which have seen significant strides forward in research, several studies have increased our understanding of lipid-derived interactions, lipid enzyme modifications, and the crucial role of lipid enzymes in defense response. First and foremost, plant cuticular waxes provide primary resistance to restrict the physical outgrowth of phytopathogens. Due to the continuous acceleration in broad-spectrum “omics” research efforts and the refinements in supportive instrumentation, we have recently acquired knowledge about the mechanism by which host plants use complex orchestrated membrane systems to utilize pathogen-derived lipids, elicitor molecules, and sphingolipids for activating signaling cascades. As a result, it is now known that plant lipids play central roles in the biosynthesis of cutin (Figure 2), the stimulation of signaling pathways that trigger different immune responses, and the reprogramming of defense-related genes.

Interestingly, in the past two decades, the identification of oxylipin’s (including JA) role in defense and other biological mechanisms has increased the knowledge pool regarding oxylipins (including JA). Most importantly, studies in model plants have helped to uncover the role of lipids and associated derivatives in regulating resistance against pathogenic microbes. Overall, in the current context of available data, it has been determined that lipids play essential roles in growth, development, and the completion of the life cycle, as well as in pathogen (or elicitor) recognition and in inciting host defense responses. Despite these significant findings, knowledge has only been gathered for the model species and prominent pathogenic microbes; however, with the advent of new pathogenic races and strains appearing in new geographical areas, part of the focus has to be primarily shifted towards new plant species and new, emerging pathogenic races. This unexplored area points towards the missing pieces in the puzzle of lipid-mediated signaling.

Moreover, the mechanism behind the interaction at surface level, lipid concentration, and lipid specificity between plants and microbes at the nano-scale is yet to be considered. Pan-transcriptome and pan-proteome analysis will help in monitoring the signaling cues and activities of interaction-related compounds from the surface level. Furthermore, considering the recent discovery of RNAi and small RNA exchange between hosts and pathogens, there is a possibility of bidirectional cross-kingdom trafficking for small lipids.

Therefore, in the future, a detailed framework can be built to elucidate the sophisticated balance between the behaviors of both pathogen and host species. These continuous efforts will allow a critical understanding of gene regulation in both pathogens and hosts and the development of fine-tuned defense strategies. Furthermore, it will establish the possibility of “inducing resistance” in crops by spraying lipid and associated molecules as biocontrol agents. Additionally, the physiological roles of lipids (other than oxylipins) and phospholipases in plant–pathogen interaction can be elucidated. This could provide novel targets for the control of the progression of plant disease. Additionally, future research can focus on whether esterified forms of PO act as a reservoir for the rapid biosynthesis/release of other oxylipins. Considering all these avenues of research, there are still multiple neglected questions in lipid signaling that can be explored in near future.

## Figures and Tables

**Table 3:** Molecular regulation and response strategies of pathogens after a successful invasion in crop plants.

Pathogenic Microbial Species	Disease	Host Species	Gene Involved	Involvement of Lipase	Response(s)	References
*Xanthomonas oryzae pv. oryzae*	Bacterial leaf blight	Rice	*Xa21*	Lipase A	Causes cell wall degradation	[88,132]
*Burkholderia glumae*	Panicle Blight	Rice	*BPR1* (Encode for Type-1 pathogenesis-related protein)	Lipase A	Affects the protein’s stability and proteolytic degradation	[115]
*Xanthomonas campestris*	Crucifer pathogen	Cabbage	*HpaR1* (GntR family transcription regulator)	Extracellular lipase	Hydrolysis of ester bonds of xylan, cell wall degradation	[133]
*Botrytis cinerea*	Grey mold, Bunch root	Strawberry, Grapes, Tomato	*Bc*TOR (*TARGET OF RAPAMYCIN*), *BBR*	Extracellular triglyceride lipase (LIP1)	Plant surface penetration	[134]
*Alternaria brassicicola*	Black spot	*Brassica* sp.; Tomato	--	Extracellular lipase	Enhances spore adhesion	[60,135]
*Fusarium graminearum*	*Fusarium* head blight	Wheat, Barley, Maize	*WFhb1-1* (Candidate gene for FHB resistance)	Extracellular Fgl1, Lip1	Degradation of plant cell wall, role in fungal nutrient acquisition	[136]
*Nectria haematococca*	Stem rot	Pea	*Fsp* (Gene responsive for *Fusarium solani* f.sp. *pisi* resistance)	Extracellular NhL1	Helps in pathogen penetration and hyphal growth	[137]
*Ustilago maydis*	Corn smut	Maize	*Rec2* *(Reduced Chloroplast coverage 2)*	Extracellular lipase	Promotes fatty acids liberation to provide a signal to infectious stage	[114]
*Magnaporthe grisea*	Rice blast infection	Rice	*LRD6-6* *(LESION MIMIC RESEMBLING 6-6)*	Intracellular lipase	Increases stored lipid degradation	[138]

## Data Availability

The data presented in this study are available in this article only.

## Abbreviations

JAZ: Jasmonate ZIM-domain, PA: Phosphatidic acid, PE: Phosphatidylethanolamine, PI: phosphatidylinositol, PG: Phosphatidylglycerol, PC: Phosphatidylcholine, MGDG: Monogalactosyldiacylglycerol, DGDG: Digalactosyldiacylglycerol, GIPC: Glycosyl inositolphosphoceramides, Cer: Ceramides, GCer: Glucosylceramides, PL: Phospholipids, SL: Sulfolipids, TAG: Triacylglycerols, GL: Galactolipids, RGAs: Resistance gene analogs, NLRs: Nucleotide-binding site leucine rich repeats, RLK: Receptor-like kinases, VLCFAs: Very long-chain fatty acids, DAG: Diacylglycerol, cAMP: Cyclic adenosine monophosphate, LPS: Lipopolysaccharides, NPCs: Non-specific phospholipase C, POs: Phyto-oxylipins, JA: Jasmonic acid, JA-Ile: (+)-7-iso-jasmonoyl-L-isoleucine, PLs: phospholipases, PKs: Protein kinases, PS: Phosphatidylserine, PtdIns (4) P: Phosphatidylinositol-4-phosphate, PtdIns (4,5) P: Phosphatidylinositol-4,5-biphosphate, PLDα1: Phospholipase D α1, DGK: Diacylglycerol kinase, FFA: Free fatty acids, DAD: Defective in anther dehiscence, pPLAs: Patatin-like proteins, CMV: *Cucumber mosaic virus*, PLP2: Proteolipid protein 2, DGK-PLC: Diacylglycerol kinase/phosphor lipase C, HR: Hypersensitive response, PAMP: Pathogen associated molecular patterns, PTI: Pattern triggered immunity, Lip A: Lipase A, PUFA/PUFA (18:2,18:3): Polyunsaturated fatty acids, MAPK: Mitogen activated protein kinases, LOXs: Lipoxygenases, ET: Ethylene, NO: Nitric oxide, SA: Salicylic acid, MiSSP7 protein: MYCORRHIZA-induced SMALL SECRECTED PROTEIN 7, SSITL: *Sclerotinia sclerotiorum* integrin-like, SP7: SECREATED PROTEIN 7, CMA: Coronamic acid, CFA: Coronafacic acid, COR: Coronatine, T3SS: Type III secretion system, PLA: Phospholipase A, PLC: Phospholipase C, PLD: Phospholipase D, ROS: Reactive oxygen species, LPL: Lysophospholipids, PLA2s: Phospholipase A2, PLP: Perforin-like proteins, pPLA 2α: Patatin-related phospholipase 2A, α-DOX: α-dioxygenase, PLC1: Phospholipase C1, LBD20: LOB DOMAIN CONTAINING PROTEIN 20, LOX10: Lipoxygenase 10, Pa-CoA: Palmitoyl-CoA, Ser: Serine, 3-ketosphi: 3-ketosphinganine, Sphi: Sphinganine, 4-Hysphi: 4-Hydroxysphinganine, Cer-1-p: Ceramide-1-phosphate, IPCer: Inositol-phosphorylceramide, Gcer: Glucosylceramide, Sphi-1-p: Sphinganine-1-phosphate, HeD: Hexadecanal, Ethp: Ethanolamine phosphate, 9-/13-HP-FA: 9- or 13-hydroperoxides fatty acids, Jas: Jasmonates, E-Hy-FA: Epoxy-hydroxy fatty acids, Keto-FA: Keto-fatty acids, LAl: Leaf aldehydes, LA: Leaf alcohols, DVE-FA: Divinylether fatty acids, Hy-FA: Hydroxy fatty acids, 16C/18C-FA: 16- and 18-carbon fatty acids, A-CoA: Acyl-Coenzyme A, ω-OH A-CoA: Acyl-CoA product bearing a terminal hydroxy group at ω-position, Di/tri-OH A-CoA: Di/tri oxidated acyl-CoA product, ω-OOH A-CoA: Acyl-CoA product bearing a terminal peroxide group at ω-position, ω-OH E-A-CoA: Expoxy acyl-CoA product bearing a terminal hydroxy group at ω-position, 20-28C A-CoA: 20-28 carbon acyl-CoA, MoGl: Monoacylglycerols, AcF: Acyl ferulate, Cut: Cutin, G3P: Glycerol-3-phosphate, Lyso-PA: 1-oleoyl-2-hydroxy-*sn*-glycero-3-phosphate, DGPP: Diacylglycerol pyrophosphate, CDP-DAG: Cytidine 5’-diphosphate diacylglycerol, ETI: Effector-triggered immunity, MAPKKK: Mitogen-activated protein kinase kinase kinase, LRR: Leucine rich repeats, AHL: Acyl-homoserine lactone, SIX: SECRETED IN XYLEM, ZIM domain: Zinc-finger inflorescence meristem) domain, FHB: *Fusarium* head blight.
